# Shared neurocognitive mechanisms of attenuating self-touch and illusory self-touch

**DOI:** 10.1093/scan/nsz002

**Published:** 2019-01-15

**Authors:** Maria Pyasik, Adriana Salatino, Dalila Burin, Anna Berti, Raffaella Ricci, Lorenzo Pia

**Affiliations:** 1 SpAtial, Motor and Bodily Awareness Research Group, Department of Psychology, University of Turin, Turin, Italy; 2 Smart-Aging Research Center, Kawashima Laboratory, Institute of Development, Aging and Cancer, Tohoku University, 4-1 Seiryocho, Aoba-ku, Sendai, Japan; 3 Neuroscience Institute of Turin, Turin, Italy

**Keywords:** body ownership, sense of agency, sensory attenuation, transcranial magnetic stimulation, supplementary motor area

## Abstract

Despite the fact that any successful achievement of willed actions necessarily entails the sense of body ownership (the feeling of owning the moving body parts), it is still unclear how this happens. To address this issue at both behavioral and neural levels, we capitalized on sensory attenuation (SA) phenomenon (a self-generated stimulus is perceived as less intense than an identical externally generated stimulus). We compared the intensity of somatosensory stimuli produced by one's own intended movements and by movements of an embodied fake hand. Then, we investigated if in these two conditions SA was equally affected by interfering with the activity of the supplementary motor area (SMA; known to be related to motor intention and SA) using single-pulse transcranial magnetic stimulation. We showed that ownership of the fake hand triggered attenuation of somatosensory stimuli generated by its movements that were comparable to the attenuation of self-generated stimuli. Furthermore, disrupting the SMA eliminated the SA effect regardless of whether it was triggered by actual participant's movements or by illusory ownership. Our findings suggest that SA triggered by body ownership relies, at least in part, on the activation of the same brain structures as SA triggered by motor-related signals.

## Introduction

Any successful achievement of goal-directed actions is subserved by a perceived causality of the self that is the feeling of intending and controlling that action in order to influence the external events. Such experience, known as `sense of agency’ (Jeannerod, 2003), is assumed to depend on signals coming from the motor system (i.e. efferent signals). Specifically, whenever we intend to achieve a willed action, the brain constructs not only a specific motor program but also predictions of the sensory consequences of the given movement. Then, predicted and actual action outcomes are compared, and the result of such comparison is fundamental not only for any online motor adjustment but also for triggering sense of agency. Specifically, the stronger is the match between predicted and actual consequences, the stronger is the experience of being the agent (Haggard and Chambon, 2012; Moore, 2016). It is worth emphasizing, however, that humans move essentially through their own body (Gallese and Sinigaglia, 2010). Hence, the body and its physical properties are crucial for interacting with the external environment (Georgieff and Jeannerod, 1998). Therefore, a full and coherent experience of being an agent is also rooted on an enduring sense that the moving body belongs to oneself. The feeling of owning one's own body, known as `body ownership’ (Gallagher, 2000), is thought to be rooted on the integration of signals coming from the sensory systems. Specifically, the stronger is the spatiotemporal correspondence among the variety of incoming signals that constantly reach the body (e.g. visual, tactile, proprioceptive, interoceptive, etc.), the higher is the feeling of own (Botvinick, 2004; Holmes and Spence, 2005; Ehrsson, 2012).

Although these considerations clearly pinpoint that body ownership must be integrated within any neurocognitive model of human's experience of willed actions, existing theories are rooted almost entirely on a variety of internal efferent signals: planning, pre-motor processing, efference copy, sensorimotor predictions and so on (Moore, 2016; Haggard, 2017). Additionally, the very few available attempts to claim that body-related signals *per se* can trigger sense of agency have provided only behavioral evidence. For instance, it has been reported that the embodiment of someone else's arm due to brain damage (Pia *et al.*, 2016) entails also sense of agency over its actions (Garbarini *et al.*, 2013; Garbarini *et al.*, 2015; Fossataro *et al.*, 2016). The same happens with an `embodied’ real-sized fake hand (Burin *et al.*, 2017c; Kilteni and Ehrsson, 2017; Burin *et al.*, 2018) or a virtual avatar (Banakou and Slater, 2014; Kokkinara *et al.*, 2016).

In the present study, we reasoned that if afferent signals toward the body could induce a sense of agency as the efferent signals toward the external world did, then both these `kinds’ of sense of agency would depend, at least in part, on the activation of the same brain structures. In order to test this hypothesis, here we investigated the impact of an altered body ownership on sense of agency within a virtual lesion approach. Specifically, we used the rubber hand illusion (RHI) paradigm to create a temporary sense of ownership of a fake hand by applying synchronous tactile stimulation to the participant's own (hidden) hand and the fake hand (Botvinick and Cohen, 1998). Then, we used sensory attenuation (SA) paradigm to measure agency. SA refers to the fact that self-produced stimuli (i.e. stimuli related to the execution of a voluntary action) are perceived as less intense in comparison with externally generated stimuli (i.e. stimuli completely unrelated one's own action) of the same intensity (Blakemore *et al.*, 1998, 2002; Burin *et al.*, 2017a). SA is thought to arise when the sensory consequence of a voluntary action matches the consequence predicted by an internal forward model and, therefore, is considered to reflect the sense of agency (Blakemore *et al.*, 1999; Borhani *et al.*, 2017; Burin, *et al.*, 2017c). We combined the RHI and the SA paradigms in order to measure the SA of the stimuli produced by an embodied fake hand and compared it with the SA of the self-generated stimuli and stimuli produced by a non-embodied fake hand (the condition that represented externally generated stimuli). Then we attempted to disrupt the SA of the stimuli generated by one's own movements and embodied fake hand's movements using single-pulse transcranial magnetic stimulation (TMS) over a brain area known to subserve the intention-programming system [i.e. the supplementary motor area (SMA; Cunnington *et al.*, 2002; Carlsen *et al.*, 2015)]. It is worth noticing that motor intention and motor awareness have been linked also to the activity of the inferior parietal lobule (Desmurget and Sirigu, 2012), rostral cingulate cortex, superior parietal cortex and insular cortex (Cunnington *et al.*, 2002). However, here we aimed at investigating whether agency triggered by body-related afferent signals and agency triggered by motor-related efferent signals share some neural signatures, rather than pinpointing all of the brain structures subserving SA or the sense of agency in general. For this reason, we capitalized on the only TMS study related to motor intention/SA, which indeed targeted the SMA (Haggard and Whitford, 2004).

We predicted that feeling of ownership of the fake hand would trigger the attenuation of sensory consequences of its movements that would be comparable to attenuation of self-generated sensory stimuli and that TMS over the SMA would similarly disrupt the SA in both conditions.

## Methods

### Participants

A total of 16 right-handed (Oldfield, 1971) healthy participants (14 females; age range, 20–37 years) with no previous history of neurological disease gave written informed consent to participate in the study approved by the Bioethical Committee of the University of Turin. The study was carried out in accordance with relevant guidelines and regulations for the protection of human participants. All participants were screened against inclusion/exclusion criteria for a safety use of TMS (Rossi *et al.*, 2009).

### Procedure

The experiment consisted of two parts in a within-subject design.

Firstly, we obtained baseline measures of sense of body ownership using the RHI paradigm and sense of agency using the SA paradigm.

Secondly, since we also aimed at measuring sense of agency over the movements of an embodied fake hand, we merged the RHI and SA paradigms in a single setup (hereinafter RHI + SA). It included the SA measurement and, in addition, the measurement of subjective ownership of the fake hand as well as subjective agency over its movements (by means of *ad hoc* questionnaires). For the RHI + SA paradigm, participants were required to have both baseline effects (i.e. RHI and SA). The initial testing included 47 participants; 34 participants (81%) had the RHI effect and 27 participants (64%) had the SA effect. A total of 20 participants displayed both effects and, therefore, were selected for the second part of the experiment (4 dropped out before the second part).

Furthermore, in the SA and RHI + SA paradigms, we used single-pulse TMS to disrupt the activity of the SMA and examined how it affected the attenuation of somatosensory stimuli produced by either one's own movement or by the movement of the embodied fake hand.

As a control condition for the TMS, in a different group of 10 participants, we administered the SA paradigm with the TMS applied over the vertex, a control site that should not be expected to induce any specific effects (Heinen *et al.*, 2011; Ricci *et al.*, 2012) (see Supplementary data).

### RHI paradigm

The baseline measure of body ownership was obtained in the RHI procedure with the vertical setting (Kalckert and Ehrsson, 2012, 2014).

Participants sat in front of a wooden box (40 × 30 × 20 cm) positioned on a table top. A life-sized model left hand (i.e. a plastic glove filled with flour) was placed on the upper shelf of the box, and the participant's left hand, wearing an identical plastic glove, was placed on the lower shelf of the box. The hands were aligned vertically (20 cm apart) and positioned congruently with respect to the participant's shoulder. The space between participant's shoulder and the fake wrist was covered by a cloth, which also covered participant's real hand, creating the impression that the fake hand was the participant's outstretched hand.

Firstly, participants, being blindfolded, were asked to indicate the perceived position of their left index finger by pointing their right index finger toward a vertical cardboard attached to the right side of the box (for six trials). The position marked on the ruler that was glued to the cardboard was averaged for six trials and referred to as pre-stimulation proprioceptive judgment. Then, the participants were instructed to always maintain their gaze on the index finger of the fake hand and remain still, while a trained experimenter delivered touches of a paintbrush to both participant's and fake hand's index finger for 2 min. There were two experimental conditions that varied according to the type of stimulation: the touches were either synchronous or asynchronous (i.e. the touches to the fake and the real hand were temporally asynchronous within a random interval of ~500–1000 ms). Immediately after the stimulation phase, the participants were again blindfolded and asked to indicate the perceived position of their left index finger on the same cardboard (six trials, the average was referred to as post-stimulation proprioceptive judgment).

At the end of each condition, participants were asked to rate their agreement with 6 statements about their experience of body ownership on a scale from −3 (representing complete disagreement) to +3 (complete agreement). The questionnaire included three real statements that described the actual illusory experience and three control statements (see Supplementary data); they were selected from a previous study (Botvinick and Cohen, 1998) and administered in randomized order.

The order of stimulation conditions (synchronous and asynchronous) was counterbalanced between participants.

### SA paradigm

In order to obtain the baseline measure of SA, we compared the subjective judgments of the intensity of somatosensory electrical stimuli, which were delivered to the participant's right hand either by the participant's own button press (executed with their left hand) or by a button press performed by a non-embodied fake hand. Single-pulse TMS over the SMA was used to interfere with the SA effect.

**Fig. 1:**
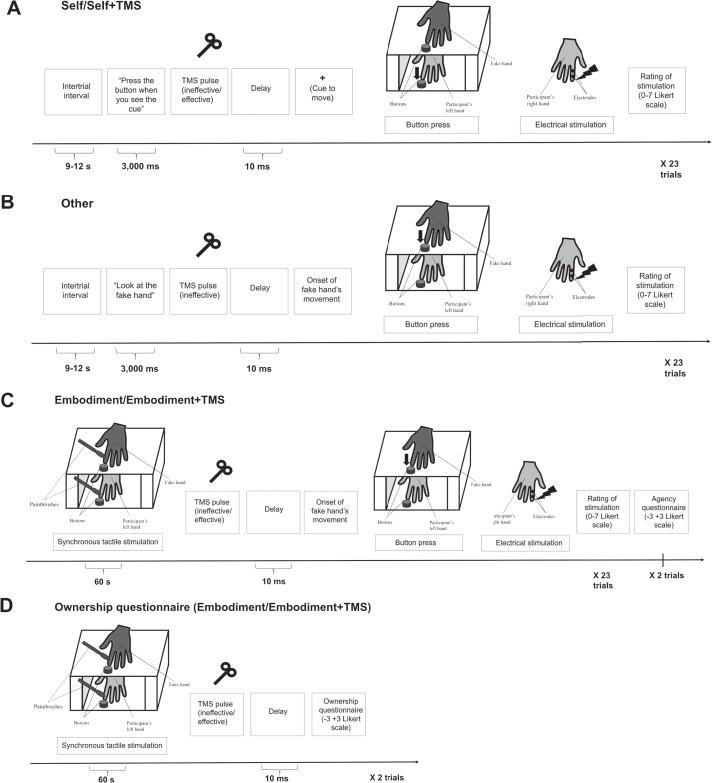
Experimental setup: timeline and procedure of a single trial in SA (**1A** and **1B**) and RHI + SA (**1C** and **1D**) paradigms.

Setup and procedure are summarized in Figure 1A and B. Here, the participants sat in front of the wooden box (same as in the RHI paradigm), with the fake hand placed on the upper shelf of the box and the participant's hand placed on the lower shelf. Additionally, they were facing a computer screen, while the TMS coil was placed on the scalp over their SMA.

TMS pulses were administered through a Magstim Rapid2 stimulator (Magstim, Whitlan, Dyfed, Wales, UK) connected to a 70 mm figure-of-eight coil positioned over the SMA. The SMA was localized at 3.3 cm anterior to Cz, according to the 10–20% system (Arai *et al.*, 2011). Before the experimental sessions, the coil was positioned over the left motor hotspot to determine the individual resting motor threshold (rMT) that was determined as the lowest stimulus intensity that induced at least three visible muscle twitches out of six consecutive TMS pulses (Rossi *et al.*, 2009). Mean rMT was 56.9 (s.d., 7.40; ranging from 42–69%) of maximum stimulator intensity.

During the experimental session, the stimulation intensity was set at 100% of the rMT, as in the previous study by Haggard and Whitford (2004). Within the experimental blocks, some trials contained `effective’ TMS pulses, while others included `ineffective’ TMS pulses (see details below). During the effective TMS trials, the coil center was placed over the SMA. In the ineffective TMS trials, the coil was positioned perpendicularly to the scalp over the SMA. Such position of the coil allows obtaining an ineffective stimulation while producing cutaneous and acoustic sensation similar to the effective TMS.

Two buttons were connected to an electrical stimulator (Digitimer DS7A); one was placed under the fake hand's index finger and the other one under participant's left index finger. The stimulator delivered electrical stimuli (2.5 subjective threshold, +4 mA with 300 V voltage) to the lateral digital nerve of the right hand by means of 5 mm diameter classical bipolar Ag/AgCl surface electrodes. The stimulation intensity was chosen according to the results of a preliminary experiment that tested the effect of different intensities (Burin *et al.*, 2017a).

The participants were instructed to maintain their gaze on a gray fixation cross on the computer screen for the intertrial interval (which varied between 9 and 12 s) and wait for instructions. Then, the fixation cross was replaced with written instructions (presented for 3 s) to either press a button upon the subsequent cue (`When you see the red cross on the screen, press the button as fast as possible’, i.e. self condition; see Figure 1B) or to look down at the index finger of the fake hand as it pressed the button (`Look at the index finger of the fake hand’, i.e. other condition; see Figure 1B). In the latter condition, the movement of the fake hand was executed by a computer-controlled servomotor that was installed inside the fake hand at the base of the index finger. In half of the trials of self condition, a single TMS pulse was delivered over the SMA (100% of rMT) 10 ms before the cue to move (i.e. self + TMS condition; see Figure 1A). The timing of the TMS pulse was chosen according to a previous study (Haggard and Whitford, 2004). In order to control for unspecific effects of TMS, single TMS pulses were also included in the remaining half of the trials in self condition and in the other condition (where it was delivered 10 ms before the onset of fake hand's movement), but, in these cases, the TMS pulses were ineffective (i.e. the coil was positioned perpendicularly to the scalp over the SMA; see Supplementary data for details). In each trial, the button press produced the somatosensory electrical stimulus to the participant's right index finger, and the participants were required to rate the perceived intensity of the stimulus on a 0–7 Likert scale (0 indicating absence of stimulation and 7 indicating the highest intensity).

The conditions were administered in a single block with randomized order of trials; each condition included 20 trials and 4 catch trials (i.e. without electrical stimulation), i.e. 72 trials in total. The position of the electrodes along the later digital nerve was changed every 7–10 trials.

### RHI + SA paradigm

Here, the RHI and SA paradigms were combined in order to measure SA of the stimuli produced by an embodied fake hand, the subjective feeling of ownership of the fake hand and agency over its movements. As in the SA paradigm, we used single-pulse TMS to interfere with the SMA activity.

Setup and procedure are summarized in Figure 1C and D. The RHI setting was the same as in the SA condition (however, here participants were not looking at the computer screen), with the TMS coil placed over the SMA.

The participants were asked to always maintain their gaze on the index finger of the fake hand. Then, in each trial, the experimenter synchronously stroked participant's and fake hand's index finger with paintbrushes (as in the RHI paradigm) for 1 min. Immediately after that, the fake hand's index finger moved to press the button that delivered a somatosensory electrical stimulus to the participant's right index finger. Then, the participants were asked to rate the intensity of the electrical stimulus on the 0–7 Likert scale (the physical intensity of the stimuli was the same as in the SA paradigm). In each trial, a TMS pulse was delivered over the SMA 10 ms before the onset of the fake hand's movement. Its intensity was set at 100% of the rMT, and the TMS pulses were either effective (embodiment + TMS condition; Figure 1C) or ineffective (embodiment condition; Fig. 1C), which was achieved with the same procedure as in the SA paradigm.

Each condition included 20 trials and 2 catch trials, with both conditions being presented in a single block with randomized order of trials (total of 42 trials). The position of the electrodes along the later digital nerve was changed every 7–10 trials.

Furthermore, each condition included a questionnaire on the subjective experience of agency. It was administered after one of the trials of embodiment condition and one of the trials of embodiment + TMS condition (the trials were selected pseudorandomly from the middle of the experimental block). The questionnaire included two real statements describing actual illusory experience of agency and two control statements; they were selected from a previous study (Kalckert and Ehrsson, 2014) and administered in randomized order (see Supplementary data). The participants had to rate their agreement with these statements on the scale from −3 (complete disagreement) to +3 (complete agreement).

**Fig. 2:**
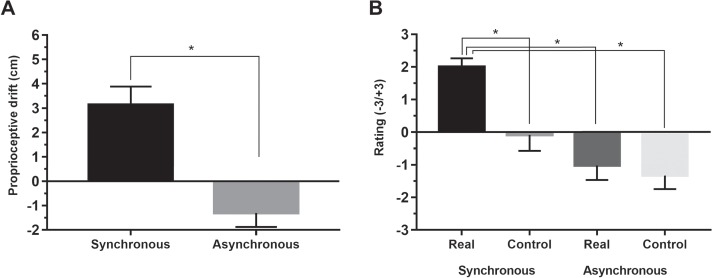
Results of RHI paradigm: **2A**, mean proprioceptive drift in synchronous and asynchronous condition; **2B**, mean ratings in real and control statements of the ownership questionnaire in synchronous and asynchronous condition. Error bars represent standard error of means; ^*^, significant.

Additionally, to control for the possible effect of TMS on the embodiment of the fake hand that was induced by the synchronous stimulation in each trial, two ownership questionnaires were included as separate trials administered pseudorandomly around the middle of the experimental block (see Figure 1D). In these trials, for 1 min, the participants experienced synchronous tactile stimulation with the paintbrushes; immediately after, a TMS pulse was delivered over the SMA, and then the questionnaire was administered. The TMS pulse was either effective (embodiment + TMS condition), or ineffective (embodiment condition). The questionnaire consisted of the same 6 statements as in the RHI paradigm, and the participants rated their agreement with the statements on the same −3 to +3 scale.

### Statistical analysis

When at least one variable in each analysis violated the criteria of normal distribution (Shapiro–Wilk test), non-parametric tests were used, and when multiple comparisons were present, the *P* values were Bonferroni corrected. The effect size was estimated using *d_z_* for parametric tests and *r* for non-parametric tests*.*

In the RHI paradigm, pre-proprioceptive judgments were subtracted from post-proprioceptive judgments and referred to as proprioceptive drift (Tsakiris and Haggard, 2005), and the ratings of real and control statements were averaged. Then, proprioceptive drift and the subjective ratings were compared between synchronous and asynchronous condition.

In the SA paradigm, stimuli intensity ratings were compared between self, other and self + TMS conditions.

In the RHI + SA paradigm, stimuli intensity ratings were firstly compared between embodiment and baseline SA conditions (self and other) then between embodiment and embodiment + TMS conditions. Ratings on real and control statements were averaged both in ownership and in agency questionnaires. Then, they were compared between embodiment and embodiment + TMS conditions.

### Data availability

All data generated or analyzed during this study are included in this article (see Supplementary data).

## Results

### RHI paradigm

According to a paired sample *t*-test, proprioceptive drift in synchronous condition (mean, 3.13 ± 3.00) was significantly higher than in asynchronous condition (mean, −1.30 ± 2.23; *t* = 4.32; df = 15; *P* < 0.001; *d_z_* = 1.64; see Figure 2A). As regards the ownership questionnaire, Wilcoxon signed-rank test showed that the ratings in real statements in synchronous condition (mean, 2.00 ± 1.05) were significantly higher than the ratings in real statements in asynchronous condition (mean, −1.02 ± 1.78; *z* = 3.21; *P* = 0.001; *r* = 0.57) and control statements in both synchronous (mean, −0.08 ± 1.95; *z* = 2.56; *P* = 0.005; *r* = 0.44) and asynchronous (mean, −1.33 ± 1.66; *z* = 3.21; *P* = 0.001; *r* = 0.57) conditions. The ratings in real statements in asynchronous condition were not significantly different from the ratings in control statements in both synchronous (*z* = 1.47; *P* = 0.14) and asynchronous (*z* = 0.31; *P* = 0.75) conditions (see Figure 2B). These results show that our sample displayed typical RHI effects in terms of both proprioceptive drift and subjective experience of ownership.

### SA paradigm

**Fig. 3:**
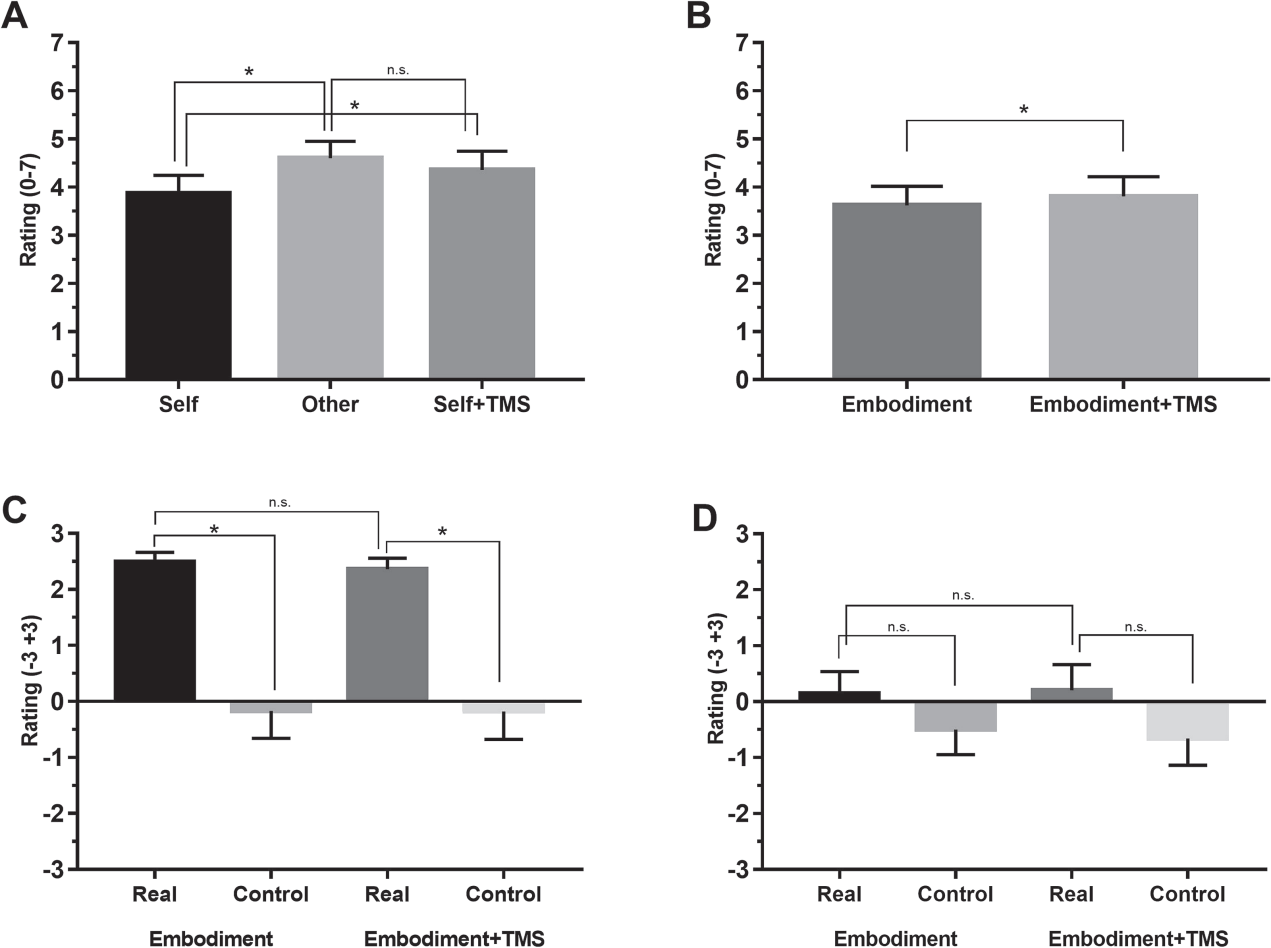
Results of SA and RHI + SA paradigm (mean ratings of the somatosensory electrical stimuli intensity): **3A**, mean intensity ratings in self, other and self + TMS conditions; **3B**, mean intensity ratings in embodiment condition compared to embodiment *+* TMS condition. Error bars represent standard error of means; ^*^, significant.

A Friedman's test for conditions (self, other and self + TMS) resulted to be significant (χ^2^ = 22.32; df = 2; *n* = 16; *P* < 0.001). A *post hoc* Wilcoxon signed-rank test showed that the ratings in self condition (mean, 3.87 ± 1.49) were significantly lower than in other (mean, 4.60 ± 1.39; *z* = 3.52; *P* < 0.001; *r* = −0.62) and in self + TMS (mean, 4.36 ± 1.55; *z* = 3.15; *P* = 0.002; *r* = −0.55), while the ratings in self + TMS were not significantly different from other (*z* = 1.91; *P* = 0.06); see Figure 3A. These results show that participants displayed typical SA effect, and TMS over the SMA disrupted this effect.

### RHI + SA paradigm

With respect to the perceived intensity of the somatosensory electrical stimulus delivered to the participant's right hand, a Friedman's test comparing intensities in the embodiment condition with those in the SA paradigm (self and other) resulted to be significant (χ^2^ = 18.32; df = 2; *n* = 16; *P* < 0.001). *Post hoc* Wilcoxon signed-rank tests showed that in embodiment (mean, 3.62 ± 1.60) condition, the perceived intensity was significantly lower than in other condition (*z* = 2.90; *P* = 0.004; *r* = −0.51) and did not differ from self condition (*z* = 0.93; *P* = 0.11). This result shows that when the embodied fake hand produces somatosensory electrical stimuli, its intensity is attenuated as the intensity of self-produced stimuli.

Furthermore, when the TMS was applied over the SMA before the onset of the fake hand's movement, according to a paired sample *t*-test, stimuli intensity ratings were significantly lower in embodiment condition compared to embodiment + TMS condition (mean, 3.81 ± 1.63; *t* = −2.49; df = 15; *P* = 0.025; *d_z_* = 0.12); see Figure 3B. Therefore, TMS over the SMA decreased SA of the stimuli produced by the embodied fake hand, similar to the condition of self-generated stimulation.

As regards subjective agency ratings, 2 × 2 repeated-measures Analysis of variance (ANOVA) for condition (embodiment, embodiment + TMS) × type (*real, control*) as within-subject factors did not show any significant differences (*f* = 0.49, *P* = 0.50). This shows that participants did not experience strong agency over the movements of the embodied fake hand, although there was a trend for it—the scores in real questions were positive in both conditions (mean, 0.14 ± 1.6 in embodiment condition and 0.20 ± 1.85 in embodiment + TMS condition). Additionally, TMS over the SMA did not affect the subjective sense of agency, since the ratings in real questions were not different between conditions (see Figure 3D).

As regards subjective ownership ratings, Wilcoxon signed-rank test showed that, in embodiment condition, the ratings in real statements (mean, 2.49 ± 0.70) were significantly higher than in control statements (mean, −0.17 ± 1.96; *z* = 3.41; *P* = 0.001; *r* = 0.60). Similarly, in embodiment + TMS condition, the ratings in real statements (mean, 2.36 ± 0.77) were significantly higher than in control statements (mean, −0.18 ± 2.01; *z* = 3.46; *P* < 0.001; *r* = 0.61). On the contrary, the ratings in real (*z* = 0.12; *P* = 0.41) or control (*z* = 3.41; *P* = 0.91) statements were not different between conditions (see Figure 3C). This shows that the TMS pulse over SMA did not affect embodiment.

## Discussion

Here we tested whether body-related signals subserving body ownership act upon agency attribution as motor-related signals are known to do. We compared attenuation of somatosensory stimuli generated by the movements of one's own hand, a fake hand and an embodied fake hand with or without TMS applied over the SMA.

Firstly, in the baseline condition, that is when body ownership (RHI) was evaluated separately from SA, our sample presented the typical RHI effects (i.e. the fake hand was embodied at both implicit and explicit levels when stimulated synchronously with the participant's own hand). These results are in line with previous literature pinpointing the importance of spatiotemporal integration of incoming sensory signals in the experience of the body as one's own (Botvinick and Cohen, 1998; Ehrsson *et al.*, 2005; Holmes and Spence, 2005; Tsakiris and Haggard, 2005; Costantini and Haggard, 2007; Longo *et al.*, 2008; Petkova *et al.*, 2011; Burin *et al.*, 2017b; Burin *et al.*, 2017c; Pyasik *et al.*, 2018). Furthermore, participants displayed the SA effect (i.e. the perceived intensity of self-produced somatosensory electrical stimuli resulted to be reduced with respect to the intensity of the same stimuli generated externally). These findings are consistent with previous data (Blakemore *et al.*, 1998; Bays *et al.*, 2005; Voss *et al.*, 2006; Voss *et al.*, 2008; Stenner *et al.*, 2014; Timm *et al.*, 2014) and further support the idea that SA is a key component of experiencing authorship over willed actions.

Secondly, when RHI and SA were combined within the same setup and the embodied fake hand delivered the stimuli, attenuation was also present and comparable with the one observed in the SA paradigm. These findings are consistent with those obtained in two previous behavioral studies (Burin *et al.*, 2018; Burin *et al.*, 2017c). In those studies, SA and subjective experience of agency were measured in three different conditions: over the movements of one's own hand, an embodied fake hand (synchronous stimulation) or a non-embodied fake hand (no stimulation/asynchronous stimulation/synchronous stimulation of incongruently located fake hand) when they pressed a button delivering a stimulus to the participant's body. As in the present study, the stimulus intensity was attenuated when one's own or the embodied fake hand delivered the stimulus but not when the non-embodied fake hand delivered the stimulus (which, in turn, meant that attenuation was not related to the kind of stimulation—synchronous or asynchronous—but rather to ownership *per se*). Considering these findings, here we administered the only condition that evoked ownership, i.e. synchronous stimulation with congruent location of the fake hand. However, contrary to those studies, here we did not find that movements of the embodied fake hand were also subjectively attributed to one's own will (explicit sense of agency), although there was a trend for it, i.e. the ratings in the questionnaire statements that described the feeling of agency were positive. Hence, one might argue that, in this case, SA alone might reflect some process unrelated to the sense of agency. Despite some authors agreed with this view (Hughes *et al.*, 2012; Dewey and Knoblich, 2014; Weller *et al.*, 2017), the majority of the existing literature pinpoints the idea that a necessary condition of SA is the presence of the signals related to the preparation of a voluntary action (Voss *et al.*, 2006, 2008; Timm *et al.*, 2014), even in the absence of a subjective explicit representation of sense of agency (e.g. Borhani et al., 2017).

Another key point to emphasize is that our setup allowed eliminating any actual or even potential efferent signals. Indeed, participants neither performed nor intended any movements, and the use of a quick, one-shot movement achieved by the fake hand's finger prevented even priming such signals. On the contrary, with the exception of Burin *et al.* (2017c), previous above-mentioned experiments entailed, in one way or another, at least some efferent signals: actual (Banakou and Slater, 2014; Kilteni and Ehrsson, 2017)/primed (Kokkinara *et al.*, 2016) movements or actual motor intentions (Garbarini *et al.*, 2013, 2015). This, in turn, suggests that those studies cannot provide unequivocal evidence of the role of body ownership *per se* in the experience of voluntary actions. In other words, the present setup, clearly allows one to provide evidence of the role of body ownership in the subjective awareness of voluntary actions independently from any kind of motor-related signals.

Thirdly, and most importantly, single-pulse TMS over the SMA disrupted somatosensory attenuation, regardless of whether the stimuli were generated by one's own movement or the movement of the embodied fake hand. Hence, we confirmed that TMS over the SMA eliminates SA of self-generated stimuli (Haggard and Whitford, 2004). Crucially, here we also showed that the SA driven by body-related signals (i.e. the embodiment of the fake hand) was affected similarly to the SA triggered by motor-related signals (i.e. one's own intended movements), which might suggest that they share at least some neural signatures. In order to exclude the possibility that our results might be related to a non-specific stimulation effect, rather than to the interference with the target area, we added a control TMS condition. We recruited a different group of participants and performed the SA baseline paradigm (i.e. the comparison between the subjective intensity of self-generated and other-generated somatosensory stimuli) with the single-pulse TMS over the vertex. The results showed that the TMS over the vertex did not modulate the SA, thus confirming that the results of the main experiment are related to the interference with the brain area involved in motor intention and SA.

In summary, by demonstrating the same TMS-driven pattern when somatosensory stimuli were generated by one's own action and by the movement of the embodied fake hand, we showed that body-related signals act upon the SA as motor-related signals do and that these two processes depend, at least in part, on the activity of the same neural structures. Interestingly, the sense of body ownership was not affected by the interference with the SMA activity, suggesting that while agency is modulated by ownership, ownership is not affected by changes in agency (`when a hand is mine, it remains mine even when I do not recognize the agency of the action’). We suggest that future studies should try explaining how the complex interplay among the variety of afferent and efferent signals contributes to the construction of a full and coherent motor consciousness.

## Supplementary Material

Supplementary Data

## References

[ref1] Arai N., Müller-Dahlhaus F., Murakami T., et al (2011). State-dependent and timing-dependent bidirectional associative plasticity in the human SMA-M1 network. Journal of Neuroscience, 31(43), 15376–15383. https://doi.org/10.1523/JNEUROSCI.2271-11.2011 22031883 10.1523/JNEUROSCI.2271-11.2011 PMC6703519

[ref2] Banakou D., Slater M. (2014). Body ownership causes illusory self-attribution of speaking and influences subsequent real speaking. Proceedings of the National Academy of Sciences of the United States of America, 111(49), 17678–17683. https://doi.org/10.1073/pnas.1414936111 25422444 10.1073/pnas.1414936111 PMC4267370

[ref3] Bays P.M., Wolpert D.M., Flanagan J.R. (2005). Perception of the consequences of self-action is temporally tuned and event driven. Current Biology, 15(12), 1125–1128. https://doi.org/10.1016/j.cub.2005.05.023 15964278 10.1016/j.cub.2005.05.023

[ref4] Blakemore S.J., Frith C.D., Wolpert D.M. (1999). Spatio-temporal prediction modulates the perception of self-produced stimuli. Journal of Cognitive Neuroscience, 11(5), 551–559. 10.1162/089892999563607. 10511643

[ref5] Blakemore S.J., Wolpert D.M., Frith C.D. (1998). Central cancellation of self-produced tickle sensation. Nature Neuroscience, 1(7), 635–640. https://doi.org/10.1038/2870 10196573 10.1038/2870

[ref6] Blakemore S.J., Wolpert D.M., Frith C.D. (2002). Abnormalities in the awareness of action. Trends in Cognitive Sciences, 6(6), 237–242. 10.1016/S1364-6613(02)01907-1. 12039604

[ref7] Borhani K., Beck B., Haggard P. (2017). Choosing, doing, and controlling: implicit sense of agency over somatosensory events. Psychological Science, 28(7), 882–893. https://doi.org/10.1177/0956797617697693 28488908 10.1177/0956797617697693

[ref8] Botvinick M. (2004). Neuroscience. Probing the neural basis of body ownership. Science, 305(5685), 782–783. DOI: 10.1126/science.1101836 15297651 10.1126/science.1101836

[ref9] Botvinick M., Cohen J. (1998). Rubber hands `feel’ touch that eyes see. Nature, 391(6669), 756. doi:10.1126/science.1101836305/5685/782 9486643 10.1038/35784

[ref10] Burin D., Battaglini A., Pia L., Falvo G., Palombella M., Salatino A. (2017a). Comparing intensities and modalities within the sensory attenuation paradigm: preliminary evidence. Journal of Advanced Research, 8(6), 649–653. https://doi.org/10.1016/j.jare.2017.08.001 28861281 10.1016/j.jare.2017.08.001 PMC5568865

[ref11] Burin D., Garbarini F., Bruno V., et al (2017b). Movements and body ownership: evidence from the rubber hand illusion after mechanical limb immobilization. Neuropsychologia, 107, 41–47. https://doi.org/10.1016/j.neuropsychologia.2017.11.004 29109038 10.1016/j.neuropsychologia.2017.11.004

[ref12] Burin D., Pyasik M., Ronga I., Cavallo M., Salatino A., Pia L. (2018). `As long as that is my hand, that willed action is mine’: timing of agency triggered by body ownership. Consciousness and Cognition, 58, 186–192. https://doi.org/10.1016/J.CONCOG.2017.12.005 29305042 10.1016/j.concog.2017.12.005

[ref13] Burin D., Pyasik M., Salatino A., Pia L. (2017c). That's my hand! Therefore, that's my willed action: how body ownership acts upon conscious awareness of willed actions. Cognition, 166, 164–173. https://doi.org/10.1016/j.cognition.2017.05.035 28577446 10.1016/j.cognition.2017.05.035

[ref14] Carlsen A.N., Eagles J.S., MacKinnon C.D. (2015). Transcranial direct current stimulation over the supplementary motor area modulates the preparatory activation level in the human motor system. Behavioural Brain Research, 279, 68–75. https://doi.org/10.1016/j.bbr.2014.11.009 25446764 10.1016/j.bbr.2014.11.009 PMC4857713

[ref15] Costantini M., Haggard P. (2007). The rubber hand illusion: sensitivity and reference frame for body ownership. Consciousness and Cognition, 16(2), 229–240. https://doi.org/10.1016/j.concog.2007.01.001 17317221 10.1016/j.concog.2007.01.001

[ref16] Cunnington R., Windischberger C., Deecke L., Moser E. (2002). The preparation and execution of self-initiated and externally-triggered movement: a study of event-related fMRI. NeuroImage, 15(2), 373–85. https://doi.org/10.1006/nimg.2001.0976 11798272 10.1006/nimg.2001.0976

[ref17] Desmurget M., Sirigu A. (2012). Conscious motor intention emerges in the inferior parietal lobule. Current Opinion in Neurobiology, 22(6), 1004–1011. https://doi.org/10.1016/j.conb.2012.06.006 22939569 10.1016/j.conb.2012.06.006

[ref18] Dewey J.A., Knoblich G. (2014). Do implicit and explicit measures of the sense of agency measure the same thing? PLoS One, 9(10). https://doi.org/10.1371/journal.pone.0110118 10.1371/journal.pone.0110118 PMC4199671 25330184

[ref19] Ehrsson H.H. (2012). The concept of body ownership and its relation to multisensory integration In: Stein B.E., editor. *The New Handbook of Multisensory Processes*, MA: MIT Press (Cambridge), 775–792.

[ref20] Ehrsson H.H., Holmes N.P., Passingham R.E. (2005). Touching a rubber hand: feeling of body ownership is associated with activity in multisensory brain areas. Journal of Neuroscience, 25(45), 10564–10573. https://doi.org/10.1523/JNEUROSCI.0800-05.2005 16280594 10.1523/JNEUROSCI.0800-05.2005 PMC1395356

[ref21] Fossataro C., Gindri P., Mezzanato T., Pia L., Garbarini F. (2016). Bodily ownership modulation in defensive responses: physiological evidence in brain-damaged patients with pathological embodiment of other's body parts. Scientific Reports, 6, 27737 https://doi.org/10.1038/srep27737 27292285 10.1038/srep27737 PMC4904197

[ref22] Gallagher S. (2000). Philosophical conceptions of the self: implications for cognitive science. Trends in Cognitive Sciences, 4(1), 14–21. 10637618 10.1016/s1364-6613(99)01417-5

[ref23] Gallese V., Sinigaglia C. (2010). The bodily self as power for action. Neuropsychologia, 48(3), 746–755. doi: 10.1016/j.neuropsychologia.2009.09.038. 19835895 10.1016/j.neuropsychologia.2009.09.038

[ref24] Garbarini F., Pia L., Piedimonte A., Rabuffetti M., Gindri P., Berti A. (2013). Embodiment of an alien hand interferes with intact-hand movements. Current Biology, 23(2), R57–8. https://doi.org/10.1016/j.cub.2012.12.003 23347936 10.1016/j.cub.2012.12.003

[ref25] Garbarini F., Rabuffetti M., Piedimonte A., Solito G., Berti A. (2015). Bimanual coupling effects during arm immobilization and passive movements. Human Movement Science, 41, 114–126. https://doi.org/10.1016/j.humov.2015.03.003 25797919 10.1016/j.humov.2015.03.003

[ref26] Georgieff N., Jeannerod M. (1998). Beyond consciousness of external reality: a `who’ system for consciousness of action and self-consciousness. Consciousness and Cognition, 7(3), 465–477. https://doi.org/10.1006/ccog.1998.0367 9787056 10.1006/ccog.1998.0367

[ref27] Haggard P (2017). Sense of agency in the human brain. Nature Reviews. Neuroscience, 18(4), 196–207. https://doi.org/10.1038/nrn.2017.14 28251993 10.1038/nrn.2017.14

[ref28] Haggard P., Chambon V. (2012). Sense of agency. Current Biology, 22(10), R390–2. https://doi.org/10.1016/j.cub.2012.02.040 22625851 10.1016/j.cub.2012.02.040

[ref29] Haggard P., Whitford B. (2004). Supplementary motor area provides an efferent signal for sensory suppression. Cognitive Brain Research, 19(1), 52–58. https://doi.org/10.1016/j.cogbrainres.2003.10.018 14972358 10.1016/j.cogbrainres.2003.10.018

[ref30] Heinen K., Ruff C.C., Bjoertomt O., et al (2011). Concurrent TMS-fMRI reveals dynamic interhemispheric influences of the right parietal cortex during exogenously cued visuospatial attention. The European Journal of Neuroscience, 33(5), 991–1000. DOI: 10.1016/j.cub.2005.08.058 21324004 10.1111/j.1460-9568.2010.07580.x PMC3437477

[ref31] Holmes N.P., Spence C. (2005). Multisensory integration: space, time and superadditivity. Current Biology, 15(18), R762–4. DOI: 10.1016/j.cub.2005.08.058 16169476 10.1016/j.cub.2005.08.058 PMC1578214

[ref32] Hughes G., Desantis A., Waszak F. (2012). Mechanisms of intentional binding and sensory attenuation: the role of temporal prediction, temporal control, identity prediction, and motor prediction. Psychological Bulletin, 139(1), 133–151. 10.1037/a0028566. 22612280

[ref33] Jeannerod M. (2003). The mechanism of self-recognition in humans. Behavioural Brain Research, 142(1–2), 1–15. https://doi.org/10.1016/S0166-4328(02)00384-4 [pii] 12798261 10.1016/s0166-4328(02)00384-4

[ref34] Kalckert A., Ehrsson H.H. (2012). Moving a rubber hand that feels like your own: a dissociation of ownership and agency. Frontiers in Human Neuroscience, 6(40). https://doi.org/10.3389/fnhum.2012.00040 10.3389/fnhum.2012.00040 PMC3303087 22435056

[ref35] Kalckert A., Ehrsson H.H. (2014). The moving rubber hand illusion revisited: comparing movements and visuotactile stimulation to induce illusory ownership. Consciousness and Cognition, 26, 117–132. https://doi.org/10.1016/j.concog.2014.02.003 24705182 10.1016/j.concog.2014.02.003

[ref36] Kilteni K., Ehrsson H.H. (2017). Body ownership determines the attenuation of self-generated tactile sensations. Proceedings of the National Academy of Sciences of the United States of America, 114(31), 8426–8431. https://doi.org/10.1073/pnas.1703347114 28716932 10.1073/pnas.1703347114 PMC5547616

[ref37] Kokkinara E., Kilteni K., Blom K.J., Slater M. (2016). First person perspective of seated participants over a walking virtual body leads to illusory agency over the walking. Scientific Reports, 6, 28879 https://doi.org/10.1038/srep28879 27364767 10.1038/srep28879 PMC4929480

[ref38] Longo M.R., Schüür F., Kammers M.P.M., Tsakiris M., Haggard P. (2008). What is embodiment? A psychometric approach. Cognition, 107(3), 978–998. https://doi.org/10.1016/j.cognition.2007.12.004 18262508 10.1016/j.cognition.2007.12.004

[ref39] Moore J.W. (2016). What is the sense of agency and why does it matter? Frontiers in Psychology, 7, 1272 https://doi.org/10.3389/fpsyg.2016.01272 27621713 10.3389/fpsyg.2016.01272 PMC5002400

[ref40] Oldfield R.C. (1971). The assessment and analysis of handedness: the Edinburgh inventory. Neuropsychologia, 9(1), 97–113. 10.1016/0028-3932(71)90067-4. 5146491

[ref41] Petkova V.I., Björnsdotter M., Gentile G., Jonsson T., Li T.Q., Ehrsson H.H. (2011). From part- to whole-body ownership in the multisensory brain. Current Biology, 21(13), 1118–1122. https://doi.org/10.1016/j.cub.2011.05.022 21683596 10.1016/j.cub.2011.05.022

[ref42] Pia L., Garbarini F., Fossataro C., Burin D., Berti A. (2016). Sensing the body, representing the body: evidence from a neurologically based delusion of body ownership. Cognitive Neuropsychology, 33 (1–2), 112–119. https://doi.org/10.1080/02643294.2016.1185404 27314302 10.1080/02643294.2016.1185404

[ref43] Pyasik M., Burin D., Pia L. (2018). On the relation between body ownership and sense of agency: a link at the level of sensory-related signals. Acta Psychologica, 185, 219–228. https://doi.org/10.1016/J.ACTPSY.2018.03.001 29533775 10.1016/j.actpsy.2018.03.001

[ref44] Ricci R., Salatino A., Li X., et al (2012). Imaging the neural mechanisms of TMS neglect-like bias in healthy volunteers with the interleaved TMS/fMRI technique: preliminary evidence. Frontiers in Human Neuroscience, 6(326), 1–13. https://doi.org/10.3389/fnhum.2012.00326 23251130 10.3389/fnhum.2012.00326 PMC3523259

[ref45] Rossi S., Hallett M., Rossini P.M., Pascual-Leone A., Safety of TMS Consensus Group (2009). Safety, ethical considerations, and application guidelines for the use of transcranial magnetic stimulation in clinical practice and research. Clinical Neurophysiology, 120(12), 2008–2039. https://doi.org/10.1016/j.clinph.2009.08.016 19833552 10.1016/j.clinph.2009.08.016 PMC3260536

[ref46] Stenner M.P., Bauer M., Sidarus N., Heinze H.J., Haggard P., Dolan R.J. (2014). Subliminal action priming modulates the perceived intensity of sensory action consequences. Cognition, 130(2), 227–235. https://doi.org/10.1016/j.cognition.2013.11.008 24333539 10.1016/j.cognition.2013.11.008 PMC3906538

[ref47] Timm J., SanMiguel I., Keil J., Schröger E., Schönwiesner M. (2014). Motor intention determines sensory attenuation of brain responses to self-initiated sounds. Journal of Cognitive Neuroscience, 26(7), 1481–9. https://doi.org/10.1162/jocn_a_00552 24392902 10.1162/jocn_a_00552

[ref48] Tsakiris M., Haggard P. (2005). The rubber hand illusion revisited: visuotactile integration and self-attribution. Journal of Experimental Psychology. Human Perception and Performance, 31(1), 80–91. http://dx.doi.org/10.1037/0096-1523.31.1.80 15709864 10.1037/0096-1523.31.1.80

[ref49] Voss M., Ingram J.N., Haggard P., Wolpert D.M. (2006). Sensorimotor attenuation by central motor command signals in the absence of movement. Nature Neuroscience, 9(1), 26–27. doi: 10.1038/nn1592 16311591 10.1038/nn1592 PMC2636578

[ref50] Voss M., Ingram J.N., Wolpert D.M., Haggard P. (2008). Mere expectation to move causes attenuation of sensory signals. PLoS One, 3(8), e2866 https://doi.org/10.1371/journal.pone.0002866 18682736 10.1371/journal.pone.0002866 PMC2478717

[ref51] Weller L., Schwarz K.A., Kunde W., Pfister R. (2017). Was it me?—Filling the interval between action and effects increases agency but not sensory attenuation. Biological Psychology, 123, 241–249. https://doi.org/10.1016/j.biopsycho.2016.12.015 28025063 10.1016/j.biopsycho.2016.12.015

