# Supplementary material for: Shared neurocognitive mechanisms of attenuating self-touch and illusory self-touch
Source: Soc Cogn Affect Neurosci. 2019 Jan 15;14(2):119–27. doi: 10.1093/scan/nsz002 (PMC6374605; doi:10.1093/scan/nsz002)
Supplement: Supplementary Data [file nsz002_supp.zip › scan-18-210-File008.docx]

**Shared neurocognitive mechanisms of attenuating self-touch and illusory self-touch**

Maria Pyasik, Adriana Salatino, Dalila Burin, Annamaria Berti, Raffaella Ricci, Lorenzo Pia

**Ownership questionnaire**

Q1: I felt as if I was looking at my own hand;

Q2: It seemed that the touches that I felt were caused by the paintbrush that was touching the fake hand;

Q3: I felt as if the fake hand was part of my body;

Q4: It appeared as if my real hand was drifting towards the fake hand;

Q5: It seemed as if the touch I was feeling came from somewhere between my own hand and the fake hand;

Q6: It felt as if my real hand was turning “rubbery”.

**Agency questionnaire**

Q1: The fake hand was obeying my will and I could make it move just like I wanted it;

Q2: I felt as if I could control movements of the fake hand;

Q3: I felt as if the fake hand was controlling my will;

Q4: I felt as if the fake hand was controlling me.

**Supplementary control experiment**

**Methods**

*Participants*

Ten right-handed (Oldfield, 1971) healthy participants (seven females, age range 20-30 years) with no previous history of neurological disease gave written informed consent to participate in the study approved by the Bioethical Committee of the University of Turin. The study was carried out in accordance with relevant guidelines and regulations for the protection of human participants. All participants were screened against inclusion/exclusion criteria for a safety use of TMS (Rossi, Hallett, Rossini, Pascual-Leone, & Safety of TMS Consensus Group, 2009).

*Procedure*

The experimental procedure was the same as in the *SA paradigm* in the main experiment. Participants were required to rate the intensity of somatosensory electrical stimuli that were delivered to their right hand either by their own button press (performed by the left hand) or by a button press performed by a non-embodied fake hand. In this case, single-pulse TMS was delivered over the vertex (the location corresponding to the Cz, according to the 10-20% system), a site that is often chosen as control site, since it should not be expected to induce any specific effects (Heinen et al., 2011; Ricci et al., 2012).

Before the experimental session, the coil was positioned over the left motor hotspot to determine the individual resting motor threshold (rMT), that was determined as the lowest stimulus intensity that induced at least three visible muscle twitches out of six consecutive TMS pulses (Rossi et al., 2009). Mean rMT was 57.4 (SD=4.81) (ranging from 49% to 64%) of maximum stimulator intensity.

During the experimental session, stimulation intensity was set at 100% of the rMT. As in the main experiment, within the experimental block, some trials contained “effective” TMS pulses, while others included “ineffective” TMS pulses. During the effective TMS trials, the coil center was placed over the vertex. In the ineffective TMS trials, the coil was positioned perpendicularly to the scalp over the vertex.

The somatosensory electrical stimuli were delivered through the buttons connected to an electrical stimulator (Digitimer DS7A). The stimulator delivered electrical stimuli (with the same intensity as in the main experiment, i.e., 2.5 subjective threshold + 4 mA with 300V voltage) to the lateral digital nerve of the right hand by means of 5-mm-diameter classical bipolar Ag/AgCl surface electrodes.

As in the *SA paradigm*, the participants were either instructed to press the button with their left index finger following the cue presented on the computer screen (*Self* and *Self+TMS* conditions) or to look at the index finger of the fake hand as it pressed the button (*Other* condition). In half of the trials of *Self* condition, a single TMS pulse was delivered over the vertex (100% of the rMT) 10 ms before the cue to move (i.e., *Self+TMS* condition); the timing of the TMS pulse was the same as in the main experiment. In order to control for the perception of the TMS pulse per se, single TMS pulse was also included in the remaining half of the trials in *Self* condition and in *Other* condition (where it was delivered 10 ms before the onset of fake-hand’s movement), but in these cases, the TMS pulses were ineffective. In each trial, the button press caused the electrical stimulus to the participant’s right index finger, and the participants were instructed to rate the perceived intensity of the stimulus on a 0-7 Likert scale (0 indicating absence of stimulation, 7 – highest intensity).

The conditions were administered in a single block with randomized order of trials; each condition included twenty trials and four catch trials (i.e. without electrical stimulation), i.e., seventy-two trials in total. The position of the electrodes along the later digital nerve was changed every 7-10 trials.

*Statistical analysis*

The stimuli intensity ratings were compared between *Self*, *Other* and *Self+TMS* conditions. Since at least one variable violated the criteria of normal distribution (Shapiro-Wilk test), we used nonparametric analysis. The p values were Bonferroni corrected for three comparisons (i.e. alpha level for statistical significance was set at p = .017).

**Results**

The results are presented in Supplementary Fig. 1. Friedman’s test for condition (*Self, Other, Self+TMS*) resulted to be significant (χ2 = 15.85, df = 2, n = 10, p<.001). Post-hoc Wilcoxon signed-rank test showed that the ratings in *Self* condition (mean=3.40±1.33) were significantly lower than in *Other* (mean=4.38±1.27; Z=2.80, p=.005, r=-.63). Moreover, the ratings in *Self+TMS* (mean=3.31±1.43) were also significantly lower than in *Other* (Z=2.80, p=.005, r=-.63). In turn, there were no significant differences between the ratings in *Self* and *Self+TMS* (Z=.24, p=.81).

These results show that the participants presented typical SA effect, which was not affected by the single-pulse TMS over the vertex.

Supplementary Figure 1 about here

**Figure legend**

**Supplementary Figure 1.** Results of SA paradigm (mean ratings of the somatosensory electrical stimuli intensity): mean intensity ratings in *Self*, *Other* and *Self+TMS* conditions. Error bars represent standard error of means; * = significant.

**References**

Heinen, K., Ruff, C. C., Bjoertomt, O., Schenkluhn, B., Bestmann, S., Blankenburg, F., … Chambers, C. D. (2011). Concurrent TMS-fMRI reveals dynamic interhemispheric influences of the right parietal cortex during exogenously cued visuospatial attention. *European Journal of Neuroscience*, *33*(5), 991–1000. https://doi.org/10.1111/j.1460-9568.2010.07580.x

Oldfield, R. C. (1971). The assessment and analysis of handedness: The Edinburgh inventory. *Neuropsychologia*, *9*(1), 97–113. http://dx.doi.org/10.1016/0028-3932(71)90067-4.

Ricci, R., Salatino, A., Li, X., Funk, A. P., Logan, S. L., Mu, Q., … George, M. S. (2012). Imaging the neural mechanisms of TMS neglect-like bias in healthy volunteers with the interleaved TMS/fMRI technique: Preliminary evidence. *Frontiers in Human Neuroscience*, *6*(326), 1–13. https://doi.org/10.3389/fnhum.2012.00326

Rossi, S., Hallett, M., Rossini, P. M., Pascual-Leone, A., & Safety of TMS Consensus Group. (2009). Safety, ethical considerations, and application guidelines for the use of transcranial magnetic stimulation in clinical practice and research. *Clinical Neurophysiology*, *120*(12), 2008–2039. https://doi.org/10.1016/j.clinph.2009.08.016
