# Supplementary figures and images for: Shared neurocognitive mechanisms of attenuating self-touch and illusory self-touch
Source: Soc Cogn Affect Neurosci. 2019 Jan 15;14(2):119–27. doi: 10.1093/scan/nsz002 (PMC6374605; doi:10.1093/scan/nsz002)

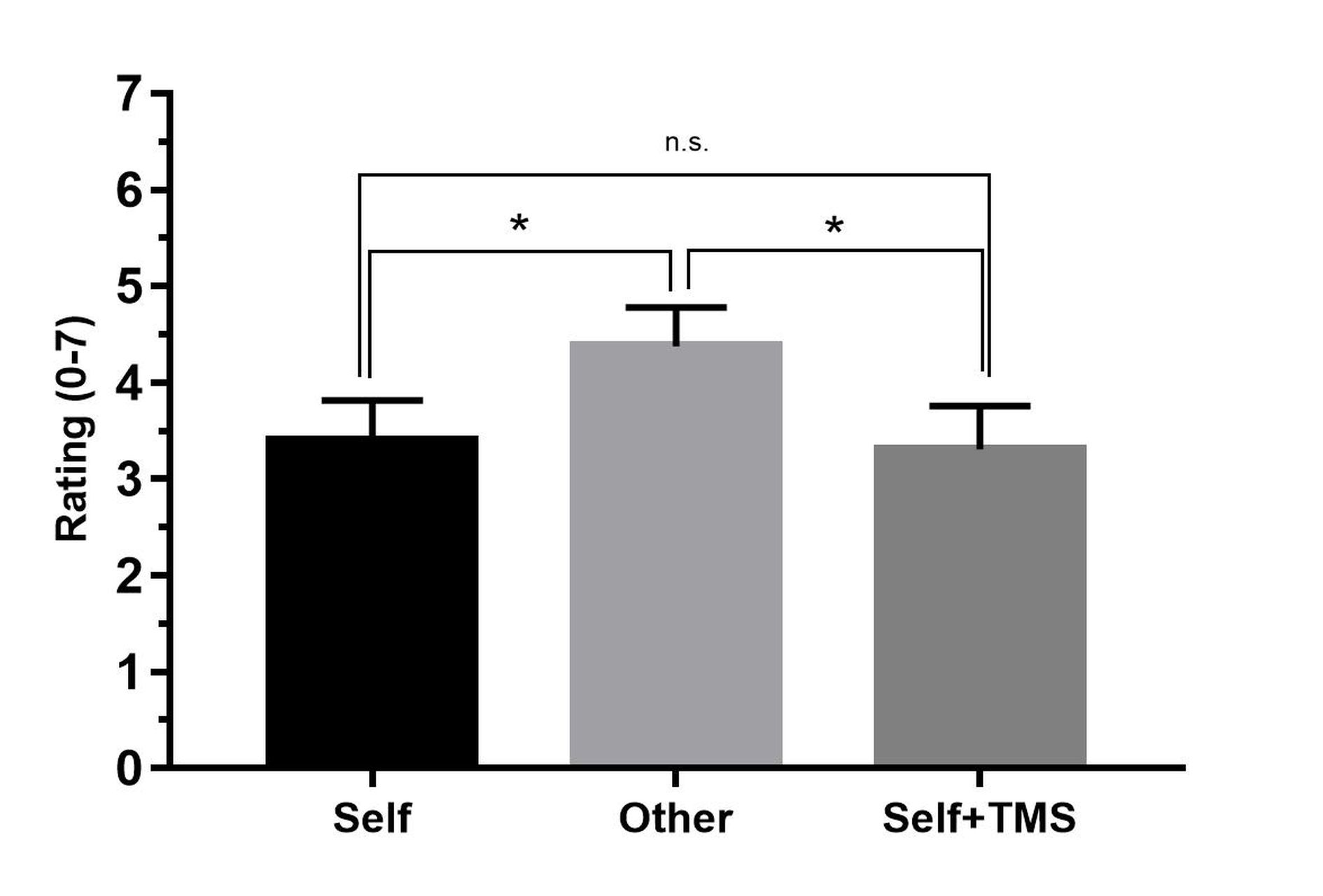

Supplement: Supplementary Data [file nsz002_supp.zip › scan-18-210-File007.jpg]
